# Author Correction: Deep mutational scanning of essential bacterial proteins can guide antibiotic development

**DOI:** 10.1038/s41467-023-36287-5

**Published:** 2023-02-06

**Authors:** Liselot Dewachter, Aaron N. Brooks, Katherine Noon, Charlotte Cialek, Alia Clark-ElSayed, Thomas Schalck, Nandini Krishnamurthy, Wim Versées, Wim Vranken, Jan Michiels

**Affiliations:** 1 Centre of Microbial and Plant Genetics, KU Leuven, Leuven, Belgium; 2 VIB-KU Leuven Center for Microbiology, Leuven, Belgium; 3 Inscripta, Inc, Boulder, CO 80301 USA; 4 Structural Biology Brussels, Vrije Universiteit Brussel, Brussels, Belgium; 5 VIB-VUB Center for Structural Biology, Brussels, Belgium; 6 Interuniversity Institute of Bioinformatics in Brussels, ULB-VUB, Brussels, Belgium

**Keywords:** Antimicrobial resistance, Bacterial genes, Antibacterial drug resistance

Correction to: *Nature Communications* 10.1038/s41467-023-35940-3, published online 16 January 2023

In this article, the affiliation VIB-VUB Center for Structural Biology, Brussels, Belgium for Wim Versées was missing. The original article has been corrected.

