# Supplementary material for: Deep mutational scanning of essential bacterial proteins can guide antibiotic development
Source: Nat Commun. 2023 Jan 16;14:241. doi: 10.1038/s41467-023-35940-3 (PMC9842644; doi:10.1038/s41467-023-35940-3)
Supplement: Supplementary file 3 — Description of Additional Supplementary Files [file 41467_2023_35940_MOESM3_ESM.pdf]

Description of Additional Supplementary Files

Supplementary Data 1: This dataset contains the read counts for all designed edits in the saturation mutagenesis libraries of *E. coli fabZ*, *lpxC* and *murA*. To verify the reproducibility of library generation, all libraries were built twice. This dataset shows the read counts of the first replicate.

Supplementary Data 2: This dataset contains the read counts for all designed edits in the saturation mutagenesis libraries of *E. coli fabZ*, *lpxC* and *murA*. To verify the reproducibility of library generation, all libraries were built twice. This dataset shows the read counts of the second replicate.

Supplementary Data 3: This dataset contains the read counts for all designed edits at several time points during a selection experiment where libraries were grown for 15 generations. This dataset was used to estimate fitness effects associated with all edits.

Supplementary Data 4: This dataset contains calculated competition coefficients that are a measure for the fitness effects associated with each edit.

Supplementary Data 5: This dataset contains the calculated tolerance score for each residue that is a measure for how tolerant each position in the tested proteins is to amino acid substitutions. This score is used to identify residues that are important for protein function (low tolerance score).

Supplementary Data 6: This dataset contains mutations that were found to provide resistance against antimicrobial compounds that target LpxC or MurA.

Supplementary Data 7: PDB files of the FabZ, LpxC and MurA proteins coloured by tolerance score are shown.
