# Supplementary material for: Deep mutational scanning of essential bacterial proteins can guide antibiotic development
Source: Nat Commun. 2023 Jan 16;14:241. doi: 10.1038/s41467-023-35940-3 (PMC9842644; doi:10.1038/s41467-023-35940-3)
Supplement: Supplementary file 11 — Reporting Summary [file 41467_2023_35940_MOESM11_ESM.pdf]

nature portfolio

Corresponding author(s): Jan Michiels, Liselot Dewachter

Last updated by author(s): Nov 18, 2022

Reporting Summary

Nature Portfolio wishes to improve the reproducibility of the work that we publish. This form provides structure for consistency and transparency in reporting. For further information on Nature Portfolio policies, see our Editorial Policies and the Editorial Policy Checklist.

Statistics

For all statistical analyses, confirm that the following items are present in the figure legend, table legend, main text, or Methods section.

n/a	Confirmed	
[ ]	[x]	The exact sample size (*n*) for each experimental group/condition, given as a discrete number and unit of measurement
[ ]	[x]	A statement on whether measurements were taken from distinct samples or whether the same sample was measured repeatedly
[x]	[ ]	The statistical test(s) used AND whether they are one- or two-sided<br>*Only common tests should be described solely by name; describe more complex techniques in the Methods section.*
[ ]	[x]	A description of all covariates tested
[ ]	[x]	A description of any assumptions or corrections, such as tests of normality and adjustment for multiple comparisons
[ ]	[x]	A full description of the statistical parameters including central tendency (e.g. means) or other basic estimates (e.g. regression coefficient) AND variation (e.g. standard deviation) or associated estimates of uncertainty (e.g. confidence intervals)
[x]	[ ]	For null hypothesis testing, the test statistic (e.g. *F*, *t*, *r*) with confidence intervals, effect sizes, degrees of freedom and *P* value noted<br>*Give P values as exact values whenever suitable.*
[x]	[ ]	For Bayesian analysis, information on the choice of priors and Markov chain Monte Carlo settings
[x]	[ ]	For hierarchical and complex designs, identification of the appropriate level for tests and full reporting of outcomes
[ ]	[x]	Estimates of effect sizes (e.g. Cohen's *d*, Pearson's *r*), indicating how they were calculated

Our web collection on statistics for biologists contains articles on many of the points above.

Software and code

Policy information about availability of computer code

Data collection	No software was used for data collection
Data analysis	The following software/code was used for data analysis: Enrich2 (version 1.3.1), BWA-MEM (version 0.7.17), R (RStudio version 1.4.1106), PyMOL(TM) Molecular Graphics System (version 2.5.0), Python (version 3.9 & 3.10.8)

For manuscripts utilizing custom algorithms or software that are central to the research but not yet described in published literature, software must be made available to editors and reviewers. We strongly encourage code deposition in a community repository (e.g. GitHub). See the Nature Portfolio guidelines for submitting code & software for further information.

Data

Policy information about availability of data

All manuscripts must include a data availability statement. This statement should provide the following information, where applicable:
- Accession codes, unique identifiers, or web links for publicly available datasets
- A description of any restrictions on data availability
- For clinical datasets or third party data, please ensure that the statement adheres to our policy

Sequencing datasets generated during the current study have been deposited in the Sequence Read Archive repository with BioProject accession number PRJNA887006 [https://www.ncbi.nlm.nih.gov/bioproject/PRJNA887006]. PDB files used in this study are FabZ FabZ PDB 6n3p [https://doi.org/10.2210/pdb6n3p/pdb], LpxC PDB 4mqy [https://doi.org/10.2210/pdb4MQY/pdb], and MurA PDB 1uae [https://doi.org/10.2210/pdb1UAE/pdb]. Read counts, other processed data resulting from our sequencing datasets and PDB files (not generated during this study) displaying tolerance scores (calculated in this study) and corresponding

March 2021

PyMol scenes are provided as Supplementary Data. The MIC and resistance data generated in this study are provided in Supplementary Data and Source Data file.

Human research participants

Policy information about studies involving human research participants and Sex and Gender in Research.

Reporting on sex and gender	NA
Population characteristics	NA
Recruitment	NA
Ethics oversight	NA

Note that full information on the approval of the study protocol must also be provided in the manuscript.

Field-specific reporting

Please select the one below that is the best fit for your research. If you are not sure, read the appropriate sections before making your selection.

☒ Life sciences ☐ Behavioural & social sciences ☐ Ecological, evolutionary & environmental sciences

For a reference copy of the document with all sections, see nature.com/documents/nr-reporting-summary-flat.pdf

Life sciences study design

All studies must disclose on these points even when the disclosure is negative.

Sample size	The largest library (MurA) contains 8349 designed edits (< 10E5). Since E. coli cultures grown overnight contain 10E8-10E9 cells (= starting point for all experiments), the theoretical coverage of each edit is higher than 1000-10.000. This coverage was deemed sufficiently high to conduct all experiments.
Data exclusions	No data were excluded from the analyses.
Replication	The reproducibility of our deep mutational scanning method (high-throughput genomic CRISPR-Cas editing) was confirmed by building 2 replicate saturation mutagenesis libraries of FabZ, LpxC and MurA and comparing read counts for each edit in both replicate libraries.<br>To estimate fitness effects, starting from the same pool, libraries were split in 3 separate cultures that were grown independently from each other to obtain 3 replicate read count values per edit that could be used to estimate fitness effects.
Randomization	NA, no experimental groups were constructed for this study.
Blinding	NA, no experimental groups were constructed for this study.

Reporting for specific materials, systems and methods

We require information from authors about some types of materials, experimental systems and methods used in many studies. Here, indicate whether each material, system or method listed is relevant to your study. If you are not sure if a list item applies to your research, read the appropriate section before selecting a response.

Materials & experimental systems

n/a	Involved in the study
☒	☐ Antibodies
☒	☐ Eukaryotic cell lines
☒	☐ Palaeontology and archaeology
☒	☐ Animals and other organisms
☒	☐ Clinical data
☒	☐ Dual use research of concern

Methods

n/a	Involved in the study
☒	☐ ChIP-seq
☒	☐ Flow cytometry
☒	☐ MRI-based neuroimaging
